# Coluber Constrictor Bite to the Eye: A Novel Case Report of a Wild Snake Bite to the Eye in North America and Review of Literature

**DOI:** 10.7759/cureus.12125

**Published:** 2020-12-17

**Authors:** Sarah Madison Duff, Andrew Bowman, Charles Richard Blake

**Affiliations:** 1 Ophthalmology, University of Florida, Gainesville, USA

**Keywords:** snake, snakebite, bite, ocular injury, open globe, black racer, coluber constrictor

## Abstract

Snake bites involving the eye are an uncommon cause of ocular trauma; herein, we present one of the few known instances of a snake bite directly to the globe, specifically by the way of a *Coluber constrictor*, also known as the “Black Racer.”

In this case report, we describe a nine-year-old girl who presented immediately following a snake bite to the right eye. The patient’s vision was unaffected despite visualized puncture wounds through the conjunctiva with associated near-total subconjunctival hemorrhage. The patient was taken to the operating room emergently for globe exploration. Circumferential peritomy and direct view to the sclera did not reveal any lacerations or puncture and subconjunctival vancomycin, gentamycin, and dexamethasone were administered intraoperatively. Post-operatively, the patient was discharged on a regimen of oral Cephalexin. Throughout multiple follow-ups, she continued to maintain excellent vision without sequelae.

Trauma to the globe via snake bite is an exceedingly rare occurrence. Upon literature review, three out of three cases involving venomous snakes resulted in “No Light Perception” vision despite anti-venom. While nonvenomous snake bites may lend a better visual outcome, if not treated properly they may also yield poor final visual potential. Methods of treatment include oral or subconjunctival antibiotic administration with or without a steroid or cycloplegic agent. All reported cases of nonvenomous cases ultimately resulted in excellent visual potential (20/40 or better) and no reports of endophthalmitis. As such, it is evident that identifying the species of snake is of the utmost importance when considering visual prognosis. Due to very few reported incidences of globe trauma via snake bite, there is no mainstay therapy for either the venomous or nonvenomous snake bite variety. Despite this, we encourage careful pursuance of the appropriate therapy on a case-by-case basis, considering operative treatment, antivenom (if necessary), and antibiotic coverage with possible cycloplegia and steroid administration.

## Introduction

Ocular injuries from snake bites are exceedingly uncommon. While snake bites elsewhere on the body have been known to cause ophthalmic manifestations, snake bites directly to the eye are extremely unusual and rarely reported. The range of complications from snake bites to the eye is largely dependent on the extent of damage and whether the snake is venomous or nonvenomous. This is the first reported case of a wild snake in North America directly biting the eye, as the four cases of penetrating globe injuries reported thus far in North America have been by pet snakes [1-4].

In the Southern United States and the State of Florida, there are six species of venomous snakes. The *Coluber constrictor priapus*, or more colloquially called the “Black Racer,” is a common nonpoisonous snake that is unlikely to cause harm to a human unless provoked. We report a nine-year-old Caucasian female who presented to us after being bitten in the eye by a Southern Black Racer.

## Case presentation

A nine-year-old Caucasian female presented to the emergency department after being bitten in the eye by a snake. She stated that she was lizard hunting with her face near the ground when a snake launched toward her face. The snake, easily identified by the patient as a “Black Racer,” bit her on the right eyelids and eye, prior to being hit away by the patient. She immediately told her father who brought her to the hospital directly.

**Figure 1:**
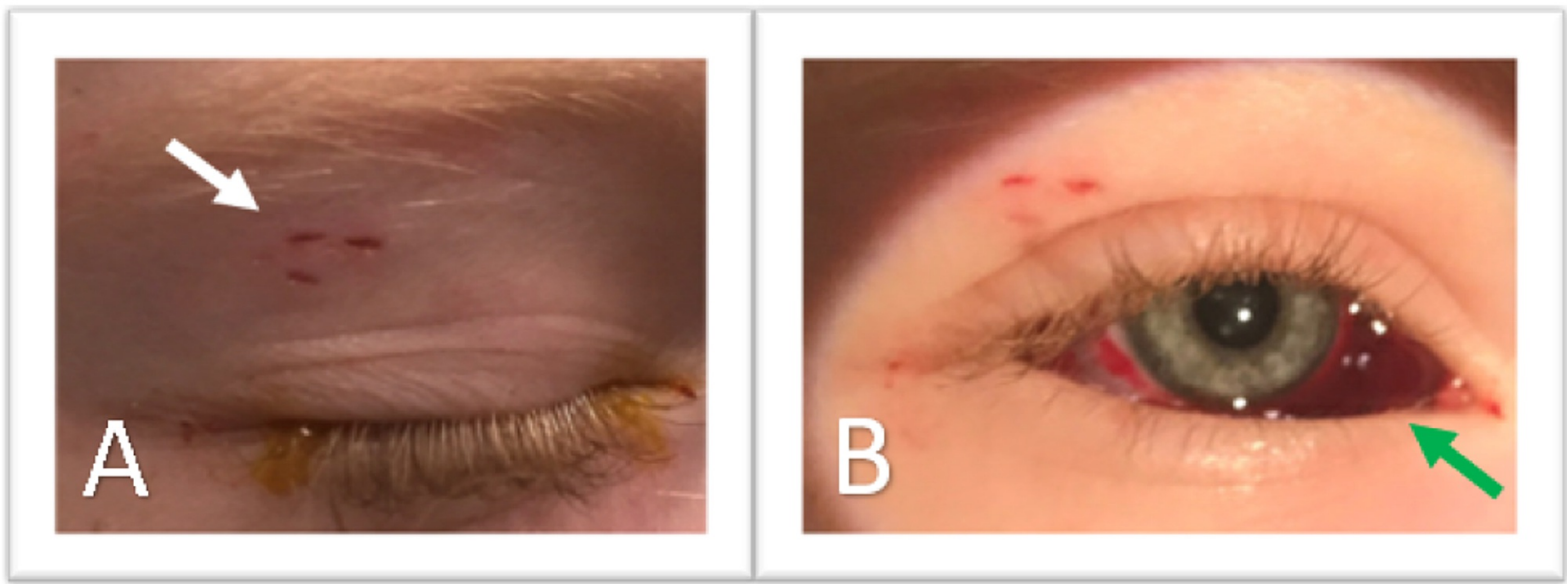
Preoperative Photographs of the Right Eye (A) Closed eyelid of the right rye showing the temporal incisions (white arrow), presumably from the fangs of the *Coluber constrictor priapus.* (B) Subconjunctival hemorrhage (green arrow) larger under the nasal bulbar conjunctiva.

On examination, the vision in both eyes was 20/20 without an affect pupillary defect (APD). Anterior chambers were deep in both eyes. Intraocular pressures were noted at 15 and 13 mm of mercury in the right and left eye, respectively, via iCare tonometer (Tiolat Oy, Helsinki, Finland). A near-total subconjunctival hemorrhage, more significant nasally, was visualized in the right eye. Puncture sites through the conjunctiva were visualized inferonasally with a few bloody tears present. The sclera could not be visualized in this area. On the upper eyelid, skin incisions from the presumed snake fangs were visualized on the lateral right eyelid (Figure 1).

The intraocular examination was normal, without vitreous hemorrhage, disc edema, retinal hemorrhages, or retinal tears. Despite the excellent vision and unconcerning intraocular pressure, there was a strong concern for sclera perforations underlying the visualized conjunctival puncture sites. With the known history of a Southern Black Racer with needle-like fangs biting the right eye, and the visualized incisions on the lateral eyebrow and conjunctiva (without direct visualization of areas of sclera covered by blood), the decision was made for an examination under anesthesia and exploration of the globe. This was to ensure that an open globe and foreign bodies were not present. Prior to transfer to the operating room, the patient received intravenous (IV) clindamycin and IV ceftriaxone.

**Figure 2:**
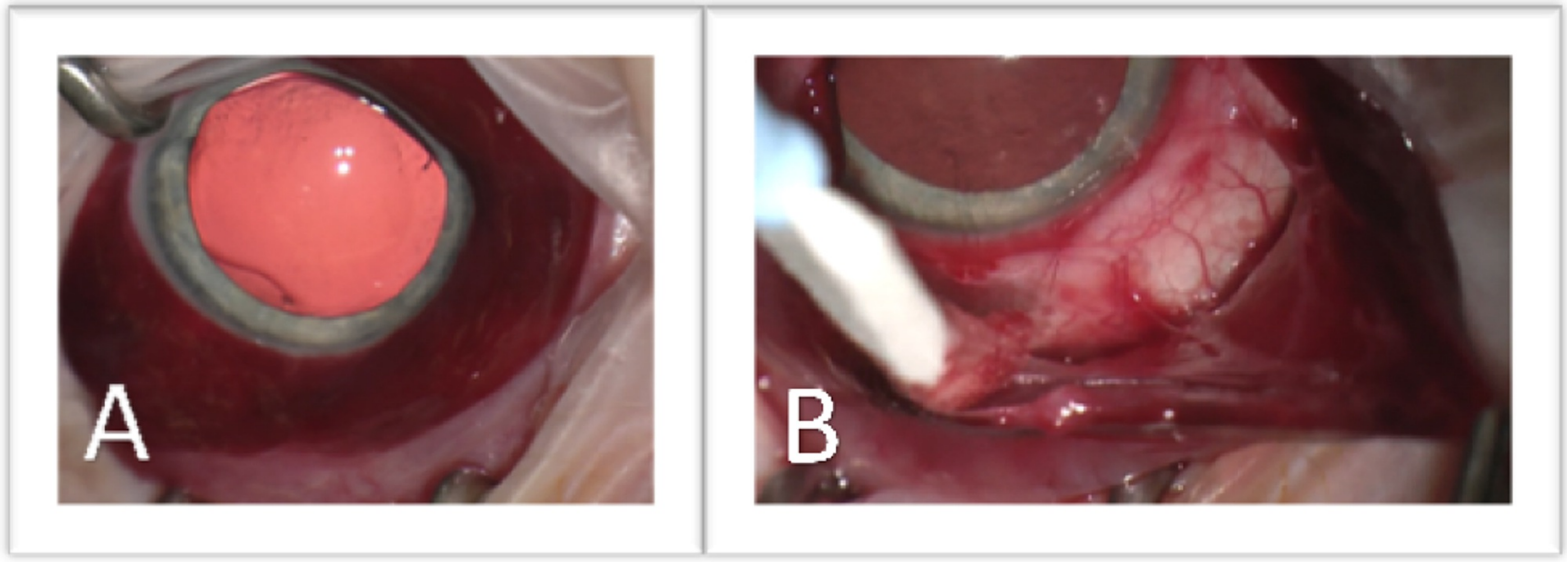
Intraoperative Photos of the Right Eye (A) Subconjunctival hemorrhage with an area of concern inferonasally. (B) Exposed sclera in the inferonasal area without the visible site of penetration.

After sedation, the right eye was examined under the operating scope and the site of the conjunctival incisions was again visualized on the nasal bulbar conjunctiva. In the setting of the circumferential subconjunctival hemorrhage, the decision was made to perform a 360-degree limbal conjunctival peritomy in order to reflect the conjunctiva and Tenon’s capsule and expose the sclera. Careful examination of the sclera, with emphasis placed on the inferior nasal sclera, did not reveal a scleral perforation or laceration, or a broken fang (Figure 2). 

The tissue was reapproximated to the limbus with 8-0 Vicryl suture and the patient was given subconjunctival injections of Vancomycin, Gentamicin, and Dexamethasone. She was discharged from the hospital with an oral course of Cephalexin. At repeat follow-ups, the patient continued to have excellent vision without concern for ocular infection. Complete healing of the conjunctiva and tenon’s capsule was found at a one-month follow-up.

## Discussion

Vision-threatening complications after a snake bite are documented, although infrequent compared to the number of snake bites worldwide each year. Most importantly in prognosis is to determine if the snake is venomous or nonvenomous. The patient may or may not be able to do this due to difficulties visualizing or identifying the snake. Venomous snake bites may cause ocular problems, even if the site of the bite is far from the patient’s eyes, or worse may cause death without treatment. Snake venom is a complex blend of proteins and peptides, unique to each species of snake. Venom effects may be differentiated into neurotoxic effects (including paralysis and pain), hemotoxic effects (including changes in blood coagulation, fibrinolysis, and platelet aggregation), and cytotoxic effects (including inflammation and necrosis) [5]. Understanding which category each snake’s venom most closely aligns with can help the physician to treat the patient. These systemic effects related to bites distant from the eyes have been reported in association with multiple diverse ocular complications, including (but not limited to): central retinal artery occlusions [6], uveitis, acute angle-closure [7], extraocular muscle palsies [8,9], optic neuritis, and optic nerve atrophy [10]. Some of these ocular complications may improve over time. In our case, the patient was able to immediately identify the snake and clarify with certainty that the snake was nonvenomous.

Direct snake bites to the eyes are equally, if not more, infrequent a cause of vision loss than snake bites elsewhere. A PubMed literature search was performed on November 6, 2020, with restrictions only for English-language articles or those translated to English, and articles with at minimum an abstract accessible through the University of Florida, using the terms “snake,” “bite,” and “eye.” Nine cases of ocular injuries from a direct snakebite injury to the eye were reported (Table 1) [1-4,11-15].

**Table 1:** Literature Review of Reported Ocular Injuries from Direct Snake Bite to the Eye VA: visual acuity, NLP: no light perception, LASIK: laser-assisted in situ keratomileusis, FabAV: fragment antigen-binding antivenom.

Authors	Year	Area	Type of Snake	Venomous (Y/N)	Wild (Y/N)	Injury	Final VA	Treatment
									Brandᾶo et al. [11]	1993	Brazil	Lance-headed Viper (Bothrops moojeni)	Yes	Yes	Periorbital swelling, exophthalmos, fang marks in the upper eyelid, necrosis of ocular tissue	NLP	Antivenom, stabilization, intravenous penicillin and ampicillin, early enucleation
Kleinman et al. [3]	1998	United States	Boa constrictor	No	No	Eyelid laceration, full-thickness corneal punctures, conjunctival puncture	20/25	Intravenous ampicillin/sulbactam, topical ofloxacin, intravenous vancomycin, ceftazidime, and clindamycin, surgical repair									
																		Sheard and Smith [12]	2003	England	Boa constrictor	No	No	Eyelid laceration, full-thickness corneal laceration, full-thickness scleral perforation	20/30	Surgical repair, topical antibiotics, systemic antibiotics, topical steroids
																											Chen et al. [13]	2003	Taiwan	Hundred pacer (Deinagkistrodon actus)	Yes	Yes	Diffuse subconjunctival hemorrhage, exophthalmos, corneal edema, full-thickness scleral laceration, necrosis of ocular tissue	NLP	D. acutus antivenom, stabilization, early evisceration
Korn and Korn [2]	2005	United States	Boa constrictor	No	No	Pinpoint area of full-thickness corneal laceration within LASIK flap	20/25	Cyanoacrylate tissue adhesive, surgical exploration, subconjunctival cefazolin and gentamicin, oral gatifloxacin, topical gatifloxacin																											
									Ashwin et al. [14]	2009	England	Python molurus	No	No	Full-thickness corneoscleral laceration, hyphema, retinal tear	20/30	Surgical repair, intravenous ceftriaxone, topical ofloxacin 0.3%, topical betamethasone 0.1%, topical cyclopentolate, argon laser retinopexy																		
																		Muthusamy and Flynn [15]	2011	England	Boa constrictor	No	No	Full-thickness corneal laceration, hyphema	20/30	Surgical repair, topical dexamethasone 0.1%, topical ofloxacin, oral ciprofloxacin, oral metronidazole									
																																				Yung et al. [4]	2018	North America	Western diamondback rattlesnake (Crotalus atrox)	Yes	No	Full-thickness scleral laceration, uveal tissue prolapse, choroidal rupture	NLP	Crotalidae FabAV, stabilization, surgical repair, intravenous levofloxacin
																											Behrens et al. [1]	2018	United States	Burmese Python (Python bivittatus)	No	No	Partial and full-thickness corneal laceration, hyphema, anterior lens capsule disruption	20/30	Intravenous cefazolin, tobramycin/dexamethasone ointment, topical moxifloxacin 0.5%, topical atropine, cataract extraction									

Of the cases in the literature, only two of the nine cases (22%) occurred in the wild [11,13], while the other cases were attacks from pets or other caged snakes. Three cases (33%) were from venomous snakes [4,11,13]. Each of these patients required anti-venom and stabilization. Despite one of these three individuals being able to physically retain his eye [4], all three cases (100%) of a venomous snake bite to the eye resulted in nonlight perception (NLP) vision or removal of the eye.

Of the nonvenomous case reports, all six cases (100%) received some form of systemic antibiotic therapy, despite no consistency in antibiotics chosen and no consistency on if intravenous or oral systemic therapy was preferred. Standard of care for nonvenomous site bites elsewhere on the body does not include empiric antibiotics to prevent secondary infections after snakebites, although tetanus prophylaxis should be provided as needed. Of those with reported drug selections (five cases) [1-3,14-15], each patient received treatment with a topical fluoroquinolone, although the specific drug of choice varied. Kleinman et al. discussed the likelihood of bacterial stomatitis, especially in captive snakes, as a reason for antibacterial therapy in ocular snakebite injuries and found that in the pet Boa constrictor case, cultures from the teeth of the snake showed multiple species of Gram-negative rods [3]. Other medications were also utilized. Three cases (50%) received a topical steroid [1,12,15], one case received subconjunctival antibiotics intraoperatively [2], and two cases (33%) received a topical cycloplegic [1,14]. Despite venomous snake bites to the eye having a poor visual prognosis, each of the nonvenomous cases ultimately ended with a best-corrected visual acuity of better than 20/40. It should be noted, however, that nonvenomous ocular snake bite cases may have had a better initial, presenting visual acuity. Regardless, none of the reported cases ultimately were reported to have endophthalmitis.

In our case, despite no reported case of endophthalmitis after a nonvenomous ocular snake bite, we treated with subconjunctival antibiotic therapy due to the concern that the fangs had penetrated the globe prior to the globe possibly self-sealing.

## Conclusions

The definitive management of a snake bite directly to the eye has yet to be fully elucidated, despite the slowly growing collection in literature. We report the first case of a wild snake biting a patient in the United States in the eye and the first reported case of a *Coluber constrictor priapus* managing to bite a patient’s eye. Despite no report of endophthalmitis after a nonvenomous ocular snake bite, we would encourage careful consideration by the practitioner prior to deferring either topical or subconjunctival antibiotic therapy. While we decided to explore the globe with surgery due to the areas of the sclera that could not be seen on examination and the knowledge that Black Racers have very long and thin fangs and use them to strike with much force and speed, due to no reports of endophthalmitis after a nonvenomous snake bite, an argument could be made to wait and watch an eye presenting similarly to ours, rather than perform an intraoperative exploration. 
